# 3,9-Dimethyl-2,3-dihydro­phenanthro[1,2-*b*]furan-4,5-dione

**DOI:** 10.1107/S1600536809047667

**Published:** 2009-11-18

**Authors:** Jing-Cai Yao, Zhong-Dong Wang, Jin-Bo Guo, Li Zhang

**Affiliations:** a College of Chemistry and Chemical Engineering, Luoyang Normal University, Luoyang 471022, People’s Republic of China; b Luoyang Zisheng Science & Technology Co Ltd, Luoyang 471000, People’s Republic of China

## Abstract

The title compound, C_18_H_14_O_3_, consists of a four-ring system which contains three six-membered rings forming a phenanthrene­dione system and a five-membered 1,2-dihydro­methyl­furan ring. A three-dimensional supra­molecular framework is formed *via* non-classical inter­molecular C—H⋯O hydrogen bonds.

## Related literature

For *tanshinone* compounds, see: Chang *et al.* (1991 ▶); Ryu *et al.* (1997 ▶); Xue *et al.* (1999 ▶); Yagi *et al.* (1989 ▶); Zhang *et al.* (2005 ▶); Zhu & Luo (2004 ▶). 
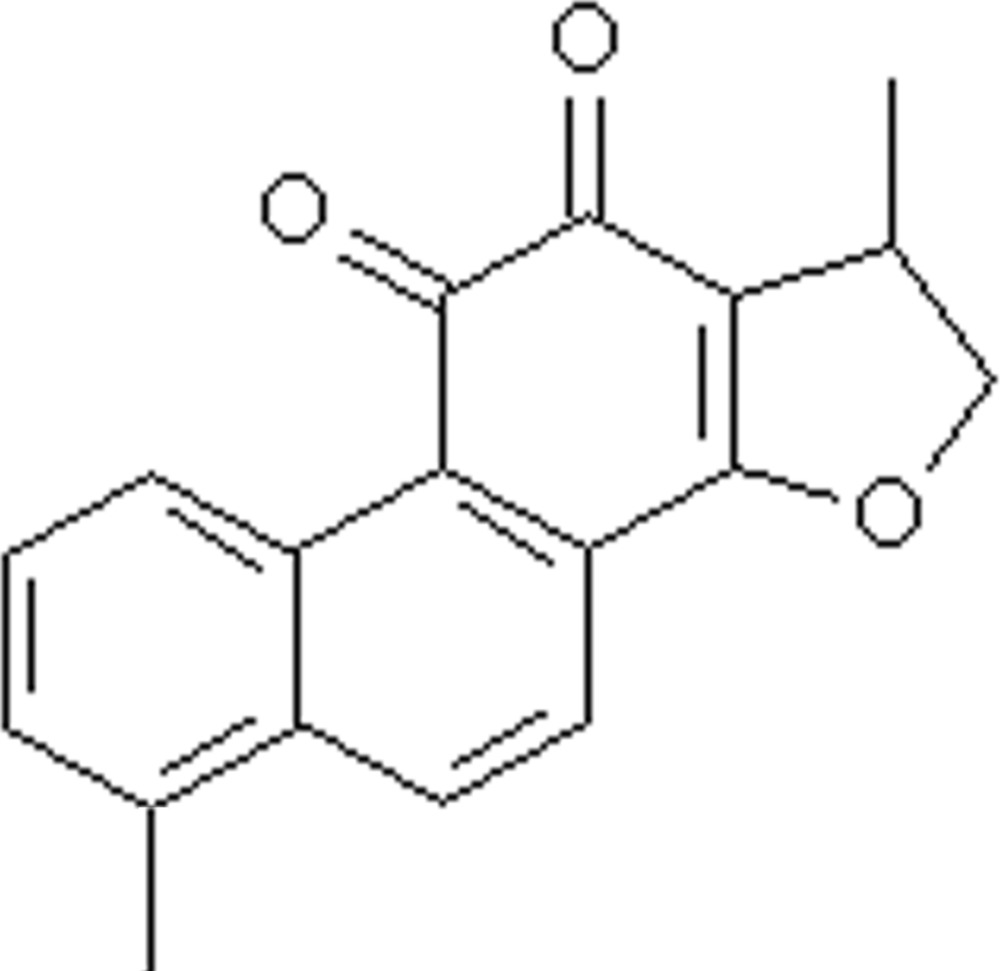


## Experimental

### 

#### Crystal data

C_18_H_14_O_3_
*M* *_r_* = 278.29
Orthorhombic, 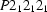

*a* = 4.6415 (10) Å
*b* = 14.692 (3) Å
*c* = 19.633 (4) Å
*V* = 1338.8 (5) Å^3^
*Z* = 4
Mo *K*α radiation
μ = 0.09 mm^−1^
*T* = 295 K
0.25 × 0.10 × 0.05 mm

#### Data collection

Bruker or SMART APEXdiffractometer
Absorption correction: multi-scan (*SADABS*; Bruker, 2004 ▶) *T*_min_ = 0.977, *T*_max_ = 0.995
6925 measured reflections
2502 independent reflections
1140 reflections with *I* > 2σ(*I*)
*R*_int_ = 0.079

#### Refinement

*R*[*F* ^2^ > 2σ(*F* ^2^)] = 0.052
*wR*(*F* ^2^) = 0.077
*S* = 1.03
2502 reflections
192 parameters
H-atom parameters constrained
Δρ_max_ = 0.38 e Å^−3^
Δρ_min_ = −0.26 e Å^−3^

### 

Data collection: *SMART* (Bruker, 2004 ▶); cell refinement: *SAINT* (Bruker, 2004 ▶); data reduction: *SAINT*; program(s) used to solve structure: *SHELXS97* (Sheldrick, 2008 ▶); program(s) used to refine structure: *SHELXL97* (Sheldrick, 2008 ▶); molecular graphics: *SHELXTL* (Sheldrick, 2008 ▶); software used to prepare material for publication: *SHELXTL*.

## Supplementary Material

Crystal structure: contains datablocks I, global. DOI: 10.1107/S1600536809047667/rk2163sup1.cif

Structure factors: contains datablocks I. DOI: 10.1107/S1600536809047667/rk2163Isup2.hkl

Additional supplementary materials:  crystallographic information; 3D view; checkCIF report

## Figures and Tables

**Table 1:** Hydrogen-bond geometry (Å, °)

*D*—H⋯*A*	*D*—H	H⋯*A*	*D*⋯*A*	*D*—H⋯*A*
C18—H18*B*⋯O1^i^	0.96	2.53	3.379 (4)	147
C16—H16*A*⋯O2^i^	0.97	2.48	3.384 (5)	155
C17—H17*A*⋯O2^ii^	0.96	2.56	3.484 (4)	162
C17—H17*C*⋯O2^iii^	0.96	2.66	3.377 (4)	132
C7—H7⋯O3^iv^	0.93	2.67	3.481 (4)	146

Symmetry codes: (i) 
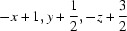
; (ii) 
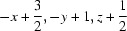
; (iii) 
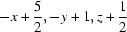
; (iv) 
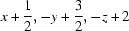
.

**Fig. 1.**
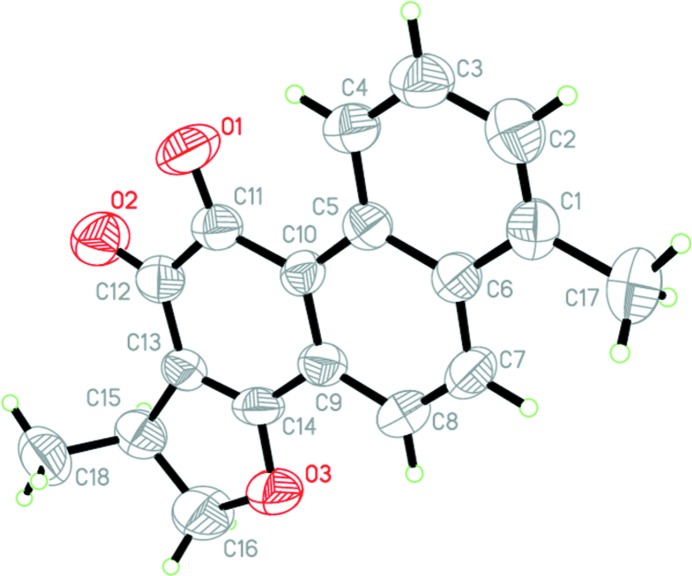
The molecular structure of I, showing the atom-numbering scheme. The displacement ellipsoids are drawn at 50% probability level. H atoms are presented as a small spheres of arbitrary radius.

**Fig. 2.**
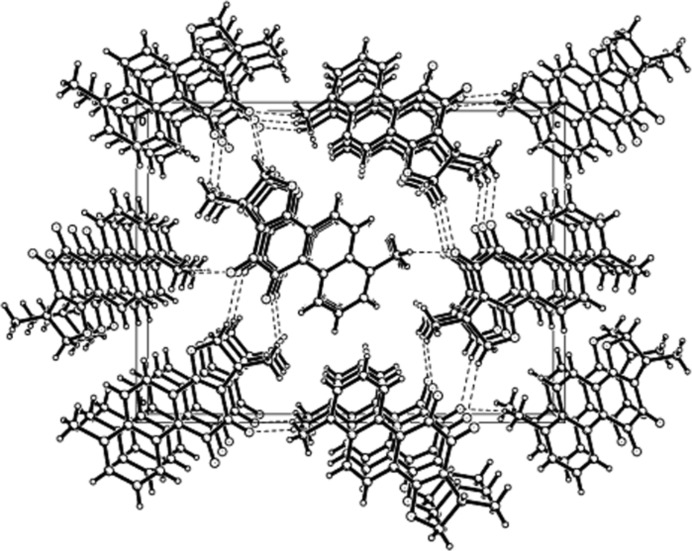
Intermolecular C—H···O non-classical hydrogen bonds and three-dimensional supramolecular framework in the crystal structure.

## supplementary crystallographic information

### Comment

The Chinese herbal medicine, *Danshen*, is the dried rhizome of *Salviae Miltiorrhiza Bunge* and *Salvia Przewalskii Maxim* (*Labiatae*). It contains two parts of efective components (liposoluble *tanshinones* and aqueous-soluble salvianolic acids). Up to date, 41 *tanshinones* and 18 salvianolic acids have been extracted and reported. *Tanshinones* have been widely used in China to treat coronary heart diseases (Chang *et al.,* 1991), antibacterial (Zhu *et al.,* 2004), antitumour (Ryu *et al.,* 1997), angina pectoris and myocardial infarction (Xue *et al.,* 1999), cerebrovascular and neurasthenic insomnia problems (Yagi *et al.,* 1989).

The title compound, C_18_H_14_O_3_, (I) consists of a four-ring system in which contains three six-membered rings forming a phenanthrene-dione system, and a five-membered 1,2-dihydro-methylfuran ring (Fig. 1). The whole molecule is essentially planar, with a torsion angle C15—C13—C14—O3 = 0.40 (4)° in the furan ring, the torsion angles C1—C2—C3—C4 = 0.01 (6)° and C4—C5—C6—C1 = -1.80 (5)° from the terminal six-membered ring. In the structure, is similar to reported (Zhang *et al.,* 2005) for 1,6-dimethylphenanthro[1,2-*b*]furan-10,11-dione, the C11—C12 bond distance agree with the corresponding distance of 1.566 (2)Å. Intermolecular C—H···O non-classical hydrogen bonds are observed in the crystal structure (Table 1), which form a three-dimensional supramolecular framework (Fig. 2).

### Experimental

All reagents were of AR grade and were used without further purification. Dried powder of Salvia miltiorrhiza Bunge was exacted with *Et*OH and the extract was concentrated in vacuo. The residue was subjected to silica gel coloumn chromatography. Elution with petroleum ether–ethyl acetate (9:1 v/v) yielded the title compound. Elemental analysis - found: C, 77.68 %; H, 5.07 %; calc. for C_18_H_14_O_3_: C, 77.14 %; H, 5.05 %.

### Refinement

All H atoms attached to C atoms were treated as riding, with C—H = 0.9300Å for aromatic H, C—H = 0.9700Å for methylene group, C—H = 0.9800Å for methyne group and C—H = 0.9600Å for methyl group with *U*_iso_(H) = 1.2*U*_eq_(C) of the carrier atoms to which they are attached and *U*_iso_(H) = 1.5*U*_eq_(C) for the methyl groups. 1004 Friedel pairs were merged.

### Crystal data

**Table:**

C_18_H_14_O_3_	*F*(000) = 584
*M**_r_* = 278.29	*D*_x_ = 1.381 Mg m^−^^3^
Orthorhombic, *P*2_1_2_1_2_1_	Mo *K*α radiation, λ = 0.71073 Å
Hall symbol: P 2ac 2ab	Cell parameters from 634 reflections
*a* = 4.6415 (10) Å	θ = 2.5–18.2°
*b* = 14.692 (3) Å	µ = 0.09 mm^−^^1^
*c* = 19.633 (4) Å	*T* = 295 K
*V* = 1338.8 (5) Å^3^	Block, yellow
*Z* = 4	0.25 × 0.10 × 0.05 mm

### Data collection

**Table:**

Bruker SMART APEX diffractometer	2502 independent reflections
Radiation source: fine-focus sealed tube	1140 reflections with *I* > 2σ(*I*)
graphite	*R*_int_ = 0.079
φ and ω scans	θ_max_ = 25.5°, θ_min_ = 2.8°
Absorption correction: multi-scan (*SADABS*; Bruker, 2004)	*h* = −5→5
*T*_min_ = 0.977, *T*_max_ = 0.995	*k* = −16→17
6925 measured reflections	*l* = −23→21

### Refinement

**Table:**

Refinement on *F*^2^	Primary atom site location: structure-invariant direct methods
Least-squares matrix: full	Secondary atom site location: difference Fourier map
*R*[*F*^2^ > 2σ(*F*^2^)] = 0.052	Hydrogen site location: inferred from neighbouring sites
*wR*(*F*^2^) = 0.077	H-atom parameters constrained
*S* = 1.03	*w* = 1/[σ^2^(*F*_o_^2^) + (0.0115*P*)^2^] where *P* = (*F*_o_^2^ + 2*F*_c_^2^)/3
2502 reflections	(Δ/σ)_max_ < 0.001
192 parameters	Δρ_max_ = 0.38 e Å^−^^3^
0 restraints	Δρ_min_ = −0.26 e Å^−^^3^

### Special details

**Table:**

Geometry. All s.u.'s (except the s.u. in the dihedral angle between two l.s. planes) are estimated using the full covariance matrix. The cell s.u.'s are taken into account individually in the estimation of s.u.'s in distances, angles and torsion angles; correlations between s.u.'s in cell parameters are only used when they are defined by crystal symmetry. An approximate (isotropic) treatment of cell s.u.'s is used for estimating s.u.'s involving l.s. planes.
Refinement. Refinement of F^2^ against ALL reflections. The weighted R-factor wR and goodness of fit S are based on F^2^, conventional R-factors R are based on F, with F set to zero for negative F^2^. The threshold expression of F^2^ > 2σ(F^2^) is used only for calculating R-factors(gt) etc. and is not relevant to the choice of reflections for refinement. R-factors based on F^2^ are statistically about twice as large as those based on F, and R-factors based on ALL data will be even larger.

### Fractional atomic coordinates and isotropic or equivalent isotropic displacement parameters (Å^2^)

**Table:**

	*x*	*y*	*z*	*U*_iso_*/*U*_eq_	
O1	0.9141 (6)	0.38978 (16)	0.80968 (12)	0.0805 (9)	
O2	0.5033 (6)	0.47076 (16)	0.73687 (12)	0.0795 (9)	
O3	0.5747 (6)	0.72625 (16)	0.87005 (12)	0.0692 (8)	
C1	1.4263 (9)	0.4722 (3)	1.04455 (18)	0.0556 (10)	
C2	1.5314 (9)	0.3891 (3)	1.03047 (19)	0.0667 (12)	
H2	1.6605	0.3621	1.0605	0.080*	
C3	1.4497 (9)	0.3423 (2)	0.9711 (2)	0.0657 (12)	
H3	1.5261	0.2849	0.9628	0.079*	
C4	1.2618 (8)	0.3789 (2)	0.92557 (19)	0.0569 (11)	
H4	1.2110	0.3464	0.8867	0.068*	
C5	1.1438 (8)	0.4660 (2)	0.93705 (17)	0.0448 (10)	
C6	1.2314 (8)	0.5139 (3)	0.99724 (17)	0.0467 (10)	
C7	1.1235 (9)	0.6017 (3)	1.00860 (17)	0.0574 (11)	
H7	1.1801	0.6331	1.0475	0.069*	
C8	0.9366 (9)	0.6424 (2)	0.96396 (18)	0.0597 (12)	
H8	0.8690	0.7009	0.9724	0.072*	
C9	0.8490 (8)	0.5959 (2)	0.90615 (18)	0.0485 (10)	
C10	0.9455 (8)	0.5084 (2)	0.89194 (16)	0.0441 (9)	
C11	0.8348 (8)	0.4632 (3)	0.82957 (18)	0.0508 (10)	
C12	0.6103 (9)	0.5125 (2)	0.78450 (18)	0.0547 (11)	
C13	0.5429 (8)	0.6037 (2)	0.80144 (18)	0.0482 (10)	
C14	0.6529 (8)	0.6393 (2)	0.8583 (2)	0.0514 (11)	
C15	0.3586 (8)	0.6724 (2)	0.76485 (18)	0.0610 (11)	
H15	0.1590	0.6507	0.7634	0.073*	
C16	0.3806 (9)	0.7536 (3)	0.81363 (19)	0.0889 (14)	
H16A	0.4581	0.8062	0.7901	0.107*	
H16B	0.1917	0.7692	0.8313	0.107*	
C17	1.5136 (8)	0.5226 (3)	1.10832 (15)	0.0772 (13)	
H17A	1.3472	0.5322	1.1365	0.116*	
H17B	1.5959	0.5803	1.0962	0.116*	
H17C	1.6532	0.4873	1.1329	0.116*	
C18	0.4609 (9)	0.6927 (2)	0.69344 (17)	0.0875 (15)	
H18A	0.6569	0.7135	0.6949	0.131*	
H18B	0.3414	0.7391	0.6737	0.131*	
H18C	0.4494	0.6385	0.6663	0.131*	

### Atomic displacement parameters (Å^2^)

**Table:**

	*U*^11^	*U*^22^	*U*^33^	*U*^12^	*U*^13^	*U*^23^
O1	0.106 (2)	0.0545 (17)	0.0810 (19)	0.0212 (18)	−0.0224 (18)	−0.0241 (15)
O2	0.096 (2)	0.0665 (18)	0.0754 (19)	−0.0015 (18)	−0.0249 (18)	−0.0080 (16)
O3	0.089 (2)	0.0457 (16)	0.0727 (18)	0.0135 (17)	−0.0073 (16)	−0.0040 (14)
C1	0.053 (3)	0.065 (3)	0.049 (3)	−0.011 (3)	0.000 (2)	−0.001 (2)
C2	0.064 (3)	0.074 (3)	0.062 (3)	0.004 (3)	−0.005 (2)	0.007 (3)
C3	0.070 (3)	0.053 (3)	0.073 (3)	0.010 (3)	−0.002 (3)	−0.002 (2)
C4	0.063 (3)	0.047 (3)	0.061 (3)	0.005 (2)	−0.001 (2)	−0.003 (2)
C5	0.044 (2)	0.044 (2)	0.047 (2)	−0.008 (2)	0.009 (2)	0.001 (2)
C6	0.047 (2)	0.047 (3)	0.045 (2)	−0.008 (2)	0.009 (2)	−0.001 (2)
C7	0.068 (3)	0.053 (3)	0.052 (3)	−0.009 (3)	0.004 (2)	−0.015 (2)
C8	0.075 (3)	0.045 (2)	0.059 (3)	0.000 (2)	0.000 (3)	−0.012 (2)
C9	0.054 (3)	0.040 (2)	0.052 (3)	−0.003 (2)	0.006 (2)	0.001 (2)
C10	0.049 (2)	0.042 (2)	0.041 (2)	−0.005 (2)	0.009 (2)	−0.004 (2)
C11	0.052 (3)	0.044 (2)	0.056 (3)	0.002 (2)	0.004 (2)	0.000 (2)
C12	0.060 (3)	0.050 (3)	0.054 (3)	−0.009 (2)	0.000 (2)	0.000 (2)
C13	0.055 (3)	0.041 (2)	0.049 (2)	−0.005 (2)	0.007 (2)	0.005 (2)
C14	0.060 (3)	0.032 (2)	0.063 (3)	0.002 (2)	0.016 (2)	0.000 (2)
C15	0.058 (3)	0.058 (3)	0.067 (3)	0.003 (2)	0.001 (2)	0.007 (2)
C16	0.111 (4)	0.065 (3)	0.091 (3)	0.029 (3)	−0.024 (3)	0.004 (3)
C17	0.078 (3)	0.099 (3)	0.055 (2)	−0.002 (3)	−0.010 (2)	−0.008 (2)
C18	0.113 (4)	0.078 (3)	0.071 (3)	0.024 (3)	0.020 (3)	0.023 (2)

### Geometric parameters (Å, °)

**Table:**

O1—C11	1.205 (4)	C8—H8	0.9300
O2—C12	1.223 (3)	C9—C10	1.390 (4)
O3—C14	1.347 (3)	C9—C14	1.455 (5)
O3—C16	1.483 (4)	C10—C11	1.485 (4)
C1—C2	1.344 (4)	C11—C12	1.547 (5)
C1—C6	1.434 (4)	C12—C13	1.416 (4)
C1—C17	1.510 (4)	C13—C14	1.335 (4)
C2—C3	1.405 (4)	C13—C15	1.505 (4)
C2—H2	0.9300	C15—C18	1.510 (4)
C3—C4	1.360 (4)	C15—C16	1.533 (4)
C3—H3	0.9300	C15—H15	0.9800
C4—C5	1.410 (4)	C16—H16A	0.9700
C4—H4	0.9300	C16—H16B	0.9700
C5—C10	1.421 (4)	C17—H17A	0.9600
C5—C6	1.435 (4)	C17—H17B	0.9600
C6—C7	1.401 (4)	C17—H17C	0.9600
C7—C8	1.371 (4)	C18—H18A	0.9600
C7—H7	0.9300	C18—H18B	0.9600
C8—C9	1.386 (4)	C18—H18C	0.9600
			
C14—O3—C16	107.0 (3)	O2—C12—C13	124.4 (4)
C2—C1—C6	118.9 (4)	O2—C12—C11	118.4 (3)
C2—C1—C17	121.2 (4)	C13—C12—C11	117.2 (4)
C6—C1—C17	119.8 (3)	C14—C13—C12	118.9 (4)
C1—C2—C3	121.2 (4)	C14—C13—C15	110.7 (3)
C1—C2—H2	119.4	C12—C13—C15	130.4 (4)
C3—C2—H2	119.4	C13—C14—O3	114.3 (4)
C4—C3—C2	121.7 (4)	C13—C14—C9	127.4 (4)
C4—C3—H3	119.2	O3—C14—C9	118.3 (4)
C2—C3—H3	119.2	C13—C15—C18	113.4 (3)
C3—C4—C5	120.2 (4)	C13—C15—C16	100.7 (3)
C3—C4—H4	119.9	C18—C15—C16	113.9 (3)
C5—C4—H4	119.9	C13—C15—H15	109.5
C4—C5—C10	123.4 (4)	C18—C15—H15	109.5
C4—C5—C6	117.9 (3)	C16—C15—H15	109.5
C10—C5—C6	118.8 (3)	O3—C16—C15	107.2 (3)
C7—C6—C1	121.0 (4)	O3—C16—H16A	110.3
C7—C6—C5	118.8 (3)	C15—C16—H16A	110.3
C1—C6—C5	120.2 (3)	O3—C16—H16B	110.3
C8—C7—C6	121.8 (4)	C15—C16—H16B	110.3
C8—C7—H7	119.1	H16A—C16—H16B	108.5
C6—C7—H7	119.1	C1—C17—H17A	109.5
C7—C8—C9	119.6 (4)	C1—C17—H17B	109.5
C7—C8—H8	120.2	H17A—C17—H17B	109.5
C9—C8—H8	120.2	C1—C17—H17C	109.5
C8—C9—C10	121.7 (4)	H17A—C17—H17C	109.5
C8—C9—C14	119.7 (4)	H17B—C17—H17C	109.5
C10—C9—C14	118.5 (4)	C15—C18—H18A	109.5
C9—C10—C5	119.3 (3)	C15—C18—H18B	109.5
C9—C10—C11	117.9 (4)	H18A—C18—H18B	109.5
C5—C10—C11	122.8 (3)	C15—C18—H18C	109.5
O1—C11—C10	124.2 (4)	H18A—C18—H18C	109.5
O1—C11—C12	116.1 (3)	H18B—C18—H18C	109.5
C10—C11—C12	119.7 (3)		
			
C6—C1—C2—C3	−1.1 (6)	C5—C10—C11—O1	−4.4 (6)
C17—C1—C2—C3	179.9 (3)	C9—C10—C11—C12	−2.3 (5)
C1—C2—C3—C4	0.0 (6)	C5—C10—C11—C12	177.7 (3)
C2—C3—C4—C5	0.2 (6)	O1—C11—C12—O2	8.4 (5)
C3—C4—C5—C10	−179.7 (3)	C10—C11—C12—O2	−173.6 (3)
C3—C4—C5—C6	0.7 (5)	O1—C11—C12—C13	−171.5 (4)
C2—C1—C6—C7	−177.9 (4)	C10—C11—C12—C13	6.5 (5)
C17—C1—C6—C7	1.1 (5)	O2—C12—C13—C14	173.8 (4)
C2—C1—C6—C5	2.0 (5)	C11—C12—C13—C14	−6.4 (5)
C17—C1—C6—C5	−179.0 (3)	O2—C12—C13—C15	−6.3 (6)
C4—C5—C6—C7	178.1 (3)	C11—C12—C13—C15	173.6 (3)
C10—C5—C6—C7	−1.5 (4)	C12—C13—C14—O3	−179.6 (3)
C4—C5—C6—C1	−1.8 (5)	C15—C13—C14—O3	0.4 (4)
C10—C5—C6—C1	178.6 (3)	C12—C13—C14—C9	2.3 (6)
C1—C6—C7—C8	−179.9 (3)	C15—C13—C14—C9	−177.7 (3)
C5—C6—C7—C8	0.2 (5)	C16—O3—C14—C13	0.8 (4)
C6—C7—C8—C9	0.5 (6)	C16—O3—C14—C9	179.1 (3)
C7—C8—C9—C10	0.1 (5)	C8—C9—C14—C13	−178.3 (4)
C7—C8—C9—C14	−179.4 (3)	C10—C9—C14—C13	2.2 (6)
C8—C9—C10—C5	−1.3 (5)	C8—C9—C14—O3	3.7 (5)
C14—C9—C10—C5	178.1 (3)	C10—C9—C14—O3	−175.8 (3)
C8—C9—C10—C11	178.7 (3)	C14—C13—C15—C18	120.7 (3)
C14—C9—C10—C11	−1.9 (5)	C12—C13—C15—C18	−59.3 (5)
C4—C5—C10—C9	−177.5 (3)	C14—C13—C15—C16	−1.4 (4)
C6—C5—C10—C9	2.0 (4)	C12—C13—C15—C16	178.7 (4)
C4—C5—C10—C11	2.5 (5)	C14—O3—C16—C15	−1.7 (4)
C6—C5—C10—C11	−178.0 (3)	C13—C15—C16—O3	1.8 (4)
C9—C10—C11—O1	175.6 (4)	C18—C15—C16—O3	−120.0 (3)

### Hydrogen-bond geometry (Å, °)

**Table:**

*D*—H···*A*	*D*—H	H···*A*	*D*···*A*	*D*—H···*A*
C18—H18B···O1^i^	0.96	2.53	3.379 (4)	147
C16—H16A···O2^i^	0.97	2.48	3.384 (5)	155
C17—H17A···O2^ii^	0.96	2.56	3.484 (4)	162
C17—H17C···O2^iii^	0.96	2.66	3.377 (4)	132
C7—H7···O3^iv^	0.93	2.67	3.481 (4)	146

Symmetry codes: (i) −*x*+1, *y*+1/2, −*z*+3/2; (ii) −*x*+3/2, −*y*+1, *z*+1/2; (iii) −*x*+5/2, −*y*+1, *z*+1/2; (iv) *x*+1/2, −*y*+3/2, −*z*+2.
